# Trammels Entailed in Convalescent Plasmapheresis During the COVID-19 Pandemic: Emphasizing the Anti-SARS-CoV-2 IgG Antibody Level in Repeat Voluntary Convalescent Plasma Donors

**DOI:** 10.7759/cureus.90826

**Published:** 2025-08-23

**Authors:** Susmita Behera, Smita Mahapatra, Madan M Majhi, Bijayalaxmi Sahoo, Nihar R Sahoo, Rabindra K Mishra

**Affiliations:** 1 Transfusion Medicine, Srirama Chandra Bhanja (SCB) Medical College and Hospital, Cuttack, IND; 2 Pathology, Srirama Chandra Bhanja (SCB) Medical College and Hospital, Cuttack, IND; 3 Community Medicine, Srirama Chandra Bhanja (SCB) Medical College and Hospital, Cuttack, IND; 4 Medicine, Maharaja Krushna Chandra Gajapati (MKCG) Medical College, Berhampur, IND; 5 Transfusion Medicine, Pandit Raghunath Murmu Medical College and Hospital, Baripada, IND

**Keywords:** antibodies, convalescent plasma, covid-19, plasma donors, plasmapheresis, sars-cov-2

## Abstract

Objective

During the outbreak of severe acute respiratory syndrome coronavirus 2, the unavailability of any specific antiviral agents or vaccines to combat the pandemic demanded an efficient therapeutic approach. Convalescent plasma (CP) emerged as an alternative therapy for COVID-19 patients. The primary objective of the study was to observe the anti-SARS-CoV-2 IgG antibody levels in repetitive convalescent plasma donation from the same individual.

Methods

The present study was carried out between 5^th^ August 2020 and 17^th^ May 2021 in the Transfusion Medicine department, Maharaja Krushna Chandra Gajapati (MKCG) Medical College, Berhampur, over nine months and 12 days.

Results

Out of the 603 individuals contacted, 564 came forward for screening, and 377 individuals donated the convalescent plasma. This study reported a majority of male donors, i.e., 355 in number. We managed to collect 37 repeat convalescent plasma units out of 400 from 14 donors. It was also observed that the maximum number of donors and recipients had the O+ve blood group. The most promising outcome of this study was to manage the convalescent plasma donation from repeat donors, i.e., five times from two plasma donors. Here, we found the declining level of antibody titer within five months between two consecutive plasma donations.

Conclusion

This study highlighted the declining anti-SARS-CoV-2 IgG antibody levels in a time-dependent manner. However, we report this as a pioneering work on repetitive convalescent plasma donation from the same individual upon following stringent inclusion and exclusion criteria to select an eligible and potential plasma donor.

## Introduction

The coronavirus disease 2019 (COVID-19) is a global health and social concern, causing public health emergencies. The epidemic spread rapidly worldwide and was characterized as a pandemic by the World Health Organization (WHO) on March 11, 2020 [1]. At that time, there were no approved specific antiviral agents targeting the novel virus, and some drugs were still under investigation. Due to the unavailability of effective vaccines and specific antiviral medicines, convalescent plasma (CP) therapy was an alternative. Convalescent plasma is the liquid component of blood enriched with antibodies and proteins collected from individuals recovered from a COVID-19 infection. This plasma contains antibodies capable of neutralizing SARS-CoV-2 antigen, potentially improving the disease progression in COVID-19 patients before their humoral response fully develops [2,3].

Convalescent plasma (CP) is among the oldest therapeutic approaches still employed during outbreaks of emerging infectious diseases. Its use dates back to the 1890s, when it was first applied in treating tetanus and diphtheria before antibiotics were developed [1]. Historical data from over 1700 cases during the 1918 Spanish influenza pandemic revealed that early administration of CP significantly reduced mortality rates. Over the past century, CP has also been utilized in managing several viral infections, such as Argentine hemorrhagic fever, Ebola, Middle East respiratory syndrome (MERS), and SARS-CoV [4]. Additionally, it has served as a preventive measure, postexposure prophylaxis, for diseases like polio, mumps, rabies, and hepatitis [5,6]. Although research on CP's effectiveness against respiratory viruses remains limited, existing evidence suggests that it works best when given early in the course of infection or used prophylactically.

The Ministry of Health and Family Welfare, Government of India, has allowed the 'off-label' use of convalescent plasma in patients with moderate COVID-19 disease who were not improving, i.e., in whom oxygen requirement was progressively increasing despite the use of steroids [7]. The US FDA approved convalescent plasma therapy as an experimental treatment for COVID-19 patients [8]. The WHO recommended strongly that COVID-19 convalescent plasma should be used in RCTs as the most effective and efficient strategy to determine the efficacy and safety of this experimental therapy [9]. So, it was necessary to identify and recruit convalescent plasma donors from the COVID-19-recovered patients. In CP donation, deferral was also a vital part of identifying eligible and potential convalescent plasma donors.

Later on, the Government of India (dated 13.06.2020) and the National Blood Transfusion Council (dated 01.07.2020) issued a circular regarding the use of convalescent plasma for moderate to severe COVID-19 patients as an off-label treatment. Then plasma therapy was started in different Medical Colleges and Hospitals of the state of Odisha as per protocol provided by the Health and Family Welfare Department, Govt. of Odisha, based on the circular of the Govt. of India.

The objective of the present study was to observe the anti-SARS-CoV-2 IgG antibody levels in repeat convalescent plasma donors (recovered from mild to moderate COVID-19 disease). We also evaluated the selection and deferral criteria for the convalescent plasma donors implemented during the plasma therapy. This study also focused on establishing the safety criteria of plasmapheresis, sharing experiences of implementing convalescent plasma therapy at Maharaja Krushna Chandra Gajapati (MKCG) Medical College and Hospital, Ganjam, Odisha, and how we managed multiple donations from single donors to meet the crisis during the peak COVID period.

## Materials and methods

Design and study population

This study was carried out for a period of nine months from 05.08.2020 to 17-05-2021 in the Transfusion Medicine Department, MKCG Medical College and Hospital (DCH), Berhampur, Odisha. The Government of Odisha established a plasma bank at MKCG MCH on August 5, 2020. A total of 603 COVID-19-recovered donors were reported at the plasma bank. Out of these, 39 persons were deferred as they didn't meet the eligibility criteria, which were set up as per the departmental standard operating procedures (SOP). A total of 564 donors were screened for plasma donation, out of which only 400 donated til the above-mentioned period.

Registry set-up

A state registry was established within the plasma bank to maintain records, and the Government of Odisha set up a public registry to enroll individuals interested in donating convalescent plasma (CP) voluntarily. A total of 603 COVID-19 recovered individuals were contacted by phone. Each potential donor was informed about the rationale for plasma donation, the detailed procedure, the duration of the procedure, the required tests to be performed, and the associated risks of the donation.

Logistic support and services to encourage voluntary plasma donation

To encourage voluntary plasma donation, the Government of Odisha offered free accommodation, food, and travel to the plasma donors traveling from nearby districts of Odisha to MKCG Medical College and Hospital, Berhampur. Upon arrival at the plasma bank, they were screened, and those who met the criteria were asked to donate their convalescent plasma (CP). Out of the 603 individuals contacted, 564 came forward for screening, and 400 individuals donated the convalescent plasma. The Government of Odisha provided certificates of appreciation to the voluntary donors to encourage their participation and felicitated them later on, in different forums.

The convalescent donors

The criteria for including and excluding donors in this clinical study were as per the guidelines laid by the Government of Odisha Health and Family Welfare Department. Donors were eligible for convalescent plasma donation if they met all of the following inclusion criteria.

Inclusion Criteria

The donor selection criteria were based on the guidelines for blood donor selection and blood donor referral. October 11, 2017, released by the National Aids Control Organization (NACO), National Blood Transfusion Council (NBTC), Government of India.

The convalescent plasma donors were required to have a prior laboratory-confirmed diagnosis of COVID-19 with complete resolution of symptoms for at least 28 days prior to donation. Additionally, a negative RT-PCR test for COVID-19 conducted at least 14 days post-recovery was mandatory. Eligible donors belonged to the 18-60-year age group and included only males or nulliparous females with a body weight exceeding 55 kg. Donors were screened for transfusion-transmitted infections such as HIV, HBV, and HCV using both enzyme-linked immunosorbent assay (ELISA) and nucleic acid amplification test (NAT), with all ELISA-negative samples also subjected to NAT as a double screening measure to ensure safe plasma for recipients. Acceptance criteria also included a total serum protein level greater than 6 g/dL and the confirmed presence of IgG antibodies to COVID-19; donors who tested negative for these antibodies were deferred. Antibody titration, aiming for a desired IgG antibody titer of 1:640, was performed through doubling dilution at the Regional Medical Research Centre (RMRC), ICMR, Bhubaneswar, with donor samples stored at temperatures below -80°C if not immediately tested. The Elecsys® anti-SARS-CoV-2 electrochemiluminescence immunoassay (ECLIA), conducted using the Abbott Architect i2000SR platform, was employed to assess antibody presence, with a reactive index ≥1.4 indicating prior exposure and a non-reactive index <1.4 signifying no detectable antibodies or exposure, thus influencing donor eligibility.

In Repeat Plasmapheresis

Donors were encouraged to donate plasma successfully. For repeat donations, the second session was arranged at least two weeks after the initial donation. Additionally, the presence of the necessary COVID-19 IgG antibody titer was checked after four weeks, ensuring that the interval between two consecutive convalescent plasma donations did not exceed four weeks. The volume of plasma collected from each donor was limited to a maximum of 500 ml per session. For the third donation within four weeks, the donor's total serum protein had to be at least 6 g/dL.

Exclusion Criteria

Individuals with uncontrolled diabetes or hypertension, pregnant women, cancer survivors, and patients with chronic conditions affecting the kidney, heart, lung, or liver are considered to be at higher risk and may require careful medical monitoring. Additionally, those with a neutralizing IgG antibody titer of less than 1:80, or an anti-SARS-CoV-2 level below 1.4 as measured by electrochemiluminescence immunoassay (ECLIA), are also categorized as vulnerable and may need additional clinical attention.

Informed consent process

The collection of convalescent plasma was conducted only after obtaining written consent from donors. Donors were informed face-to-face about the procedure's length, the volume of plasma to be collected (which would be split into two portions), and that their plasma would be used to treat two COVID-19 patients. Before this, thorough counseling was provided through both telephone calls and in-person discussions. All consent-related documentation was carefully recorded and securely stored. The complete blood count (CBC) was performed, including measurements of hemoglobin (Hb), hematocrit, platelet count, and both total and differential white blood cell counts. Successful plasmapheresis donors were requested to repeat the donation. If the donor agreed, such donations were scheduled after at least two weeks of the first plasma donation.

Acquisition and plasma composition

Convalescent plasma was obtained through plasmapheresis using the Trima Accel machine (Terumo Penpol, Kerala, India), with volumes ranging from 400 ml to a maximum of 500 ml. The collected plasma was divided into two portions, each containing between 200 and 250 ml, irrespective of the donor's age or weight. Plasma units were stored in a deep freezer at -40°C and thawed at 37°C before issue, after performing minor cross-matching. This protocol followed the guidelines issued by the Directorate General of Health Services (DGHS) and the Central Drugs Standard Control Organization (CDSCO), Government of India, on April 17, 2020, which limited plasma collection to 500 ml per session as per the Drugs and Cosmetics (Second Amendment) Rules, 2020. Donors showed no signs of infection and had tested negative for COVID-19 at least 14 days post-recovery. At the start of the pandemic, India's government established a plasma collection protocol permitting donations only after 14 days of recovery, provided the donor had a negative reverse transcription polymerase chain reaction (RT-PCR) test before donation [10]. Both RT-PCR and antibody tests for COVID-19 were required to be repeated 48 hours later and again on the day of donation [11]. Beyond medical screening, attention was also given to the donor's psychological well-being to avoid exploitation and address any vulnerabilities [12].

Infusion of blood products

The transfusions were done using blood transfusion sets as per the NBCT guidelines, 2017. An ABO-compatible plasma (200ml) was issued after minor cross-matching and thawing at 37 ⁰C. After the initial plasma transfusion, one or two additional 200 ml doses were given at 24-hour intervals based on the patient's condition and how well they tolerated the treatment. When possible, the second plasma unit was obtained from a different donor, provided another ABO-compatible unit was available.

Record keeping and reporting

All donor, kit, equipment, and recipient-related adverse events were recorded and reported. The data was then entered into an electronic system. All source documents, including clinical reports and records necessary for evaluation, were securely stored, with an emphasis on maintaining donor confidentiality. 

## Results

This cross-sectional analysis was conducted on blood donor data collected from 05th August 2020 to 18th May 2021 at MKCG MCH, Ganjam. The reasons for the deferral of donors were examined across different categories, various age groups, and whether the deferral was temporary or permanent. This study also examined the infection rates of the SARS-CoV-2 virus across different genders, age groups, and blood groups.

A total of 603 donors reported at the Plasma Bank of MKCG MCH, Ganjam, Odisha, for voluntary donations post-recovery of COVID-19. Based on the inclusion criteria mentioned in the standard operating procedures (SOP), 564 donors were eligible for plasma donation, whereas 39 donors deferred based on their medical history and physical examination (Table 1).

**Table 1:** Deferral during screening at the plasma bank

Sl No.	Cause of deferral	No. of patients (n=39)
1	Collapsed vein	17
2	Unwilling to donate at a blood bank after knowing the detailed procedure	4
3	Under thyroid medication	3
4	Uncontrolled diabetes on insulin treatment	3
5	Reported before 14 days after discharge from the hospital	3
6	Recent blood donation (within 15 days) before their COVID test	3
7	H/O asthma under medication	2
8	H/O viral hepatitis (HCV)	1
9	Hansen's disease under medication	1
10	Fractured tibia	1
11	H/O heart disease	1

The major cause of deferral was the presence of collapsed veins (n=17) in the recovered patients. Some patients (n=4) also denied donation after getting detailed information about the procedure. However, patients who were also diagnosed with hypothyroidism (n=3), uncontrolled diabetes (n=3), and asthma under medication (n=2) were deferred for plasma donation. Patients reported before 14 days after discharge (n=3) from COVID-19 recovery also deferred as per the US Food and Drug Administration regulations. Apart from these deferral criteria, also include the patients who had donated blood within 15 days (n=3). Other causes of deferral were viral hepatitis (n=1), Hansen's disease under medication (n=1), and a fractured tibia (n=1), each contributing for one patient.

**Figure 1:**
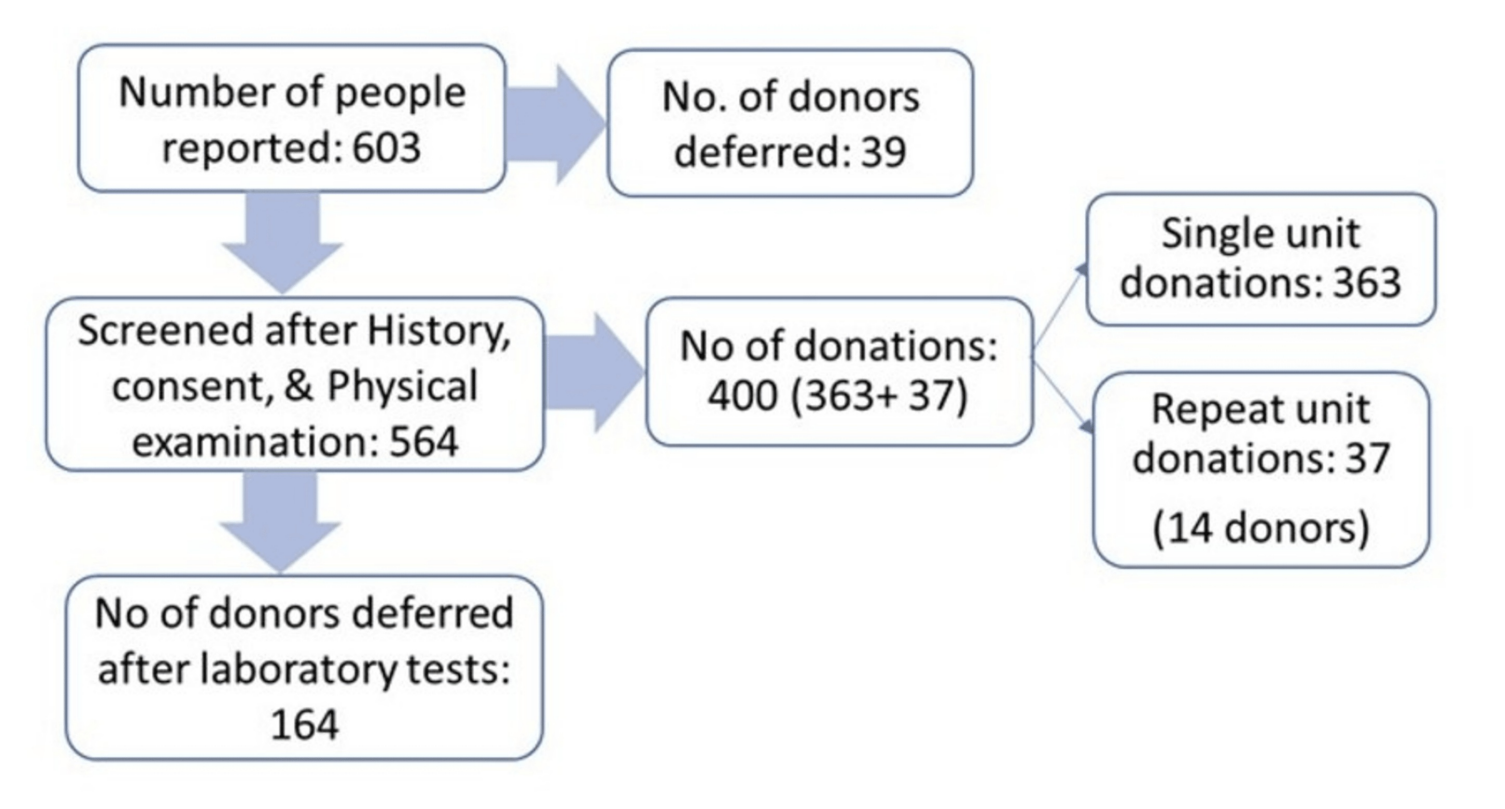
Detailed list of the number of plasma donors screened and deferred after testing

After physical examination, 564 convalescent plasma donors underwent SOP-based screening, and 164 donors were excluded based on laboratory test results (Figure 1). Sixty-four donors were deferred due to negative anti-COVID-19 IgG or titer values. Donors with HbsAg-positive results (n=21) were also excluded from donations. Some donors were deferred due to low hemoglobin (n=13) and high hematocrit (n=5) values. Twenty-five people were also denied plasma donations after the completion of screening procedures. Thirty-six donors did not respond to repetitive telephonic calls for donation and failed to report in time, and were thereby included in the deferred list of donors.

**Figure 2:**
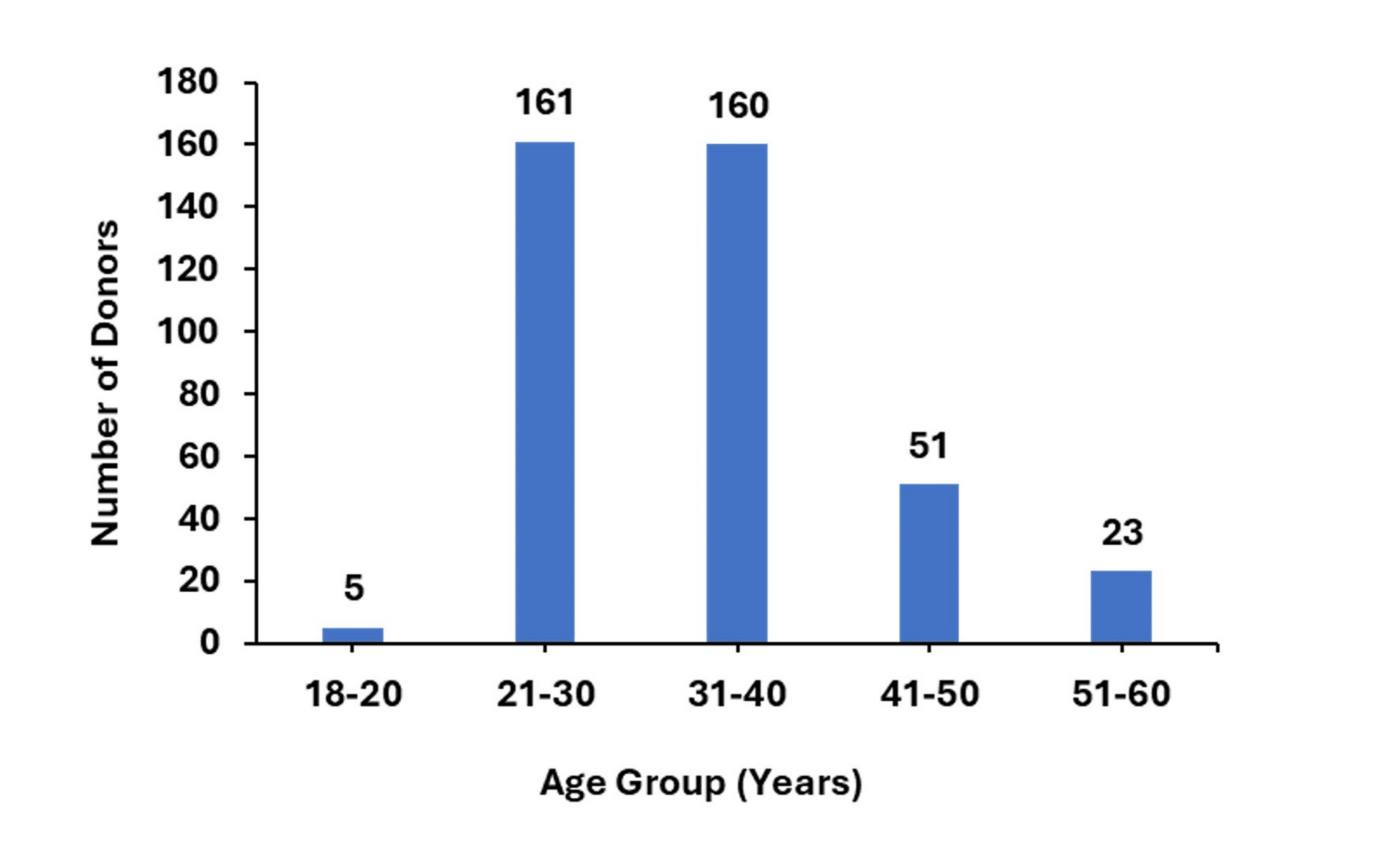
The distribution of convalescent plasma donors across different age groups

According to the data presented in Figure 2, the majority of donors who donated plasma after recovering from COVID-19 were in the younger age groups, specifically those aged between 21 and 40 years. The highest number of donations came from individuals in this age group, with up to 161 plasma units. In contrast, the least number of plasma units was collected from donors aged between 18 and 20 years.

**Figure 3:**
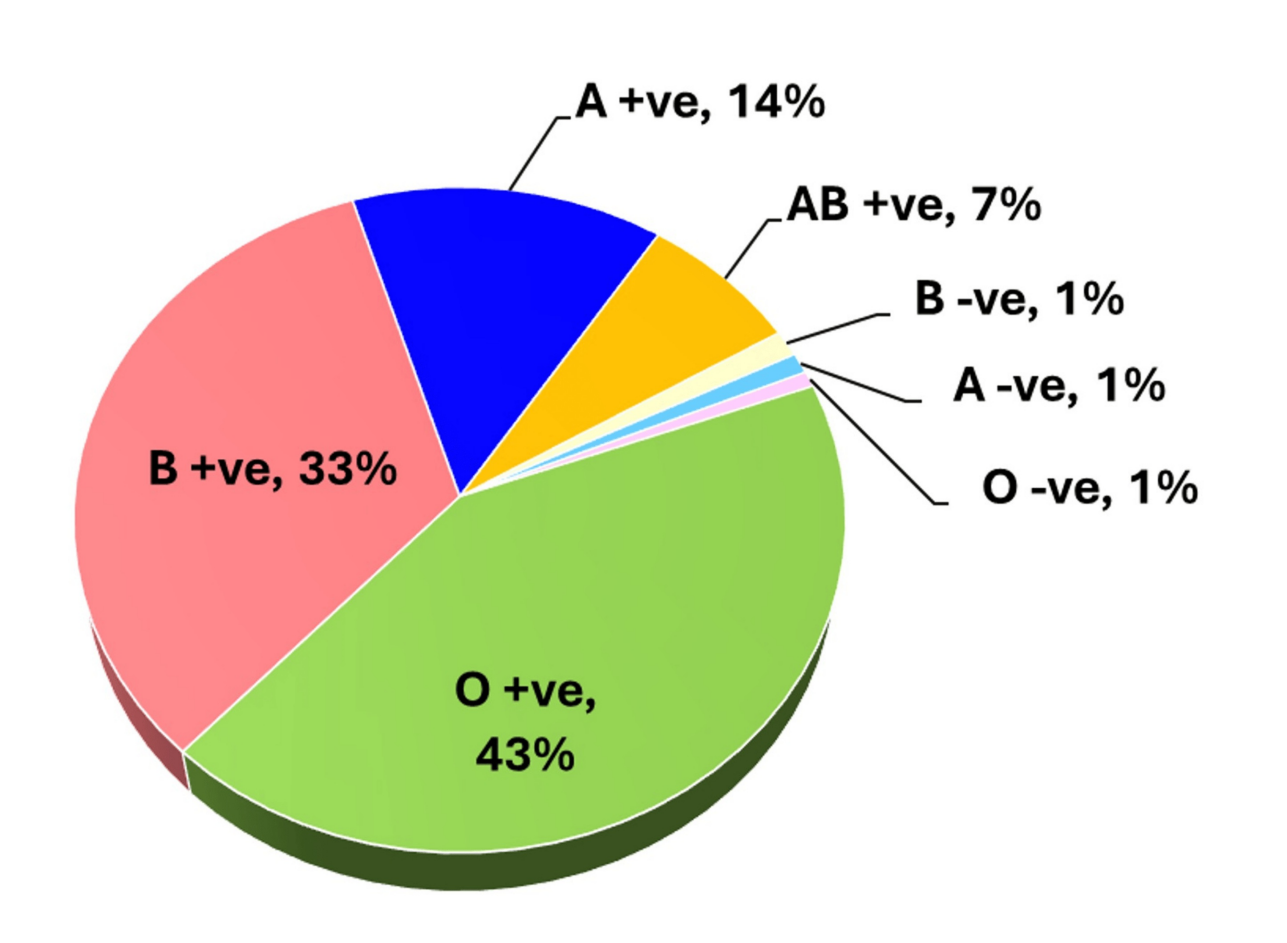
Pie chart representing the distribution of convalescent plasma donors across different blood groups

In terms of blood group typing, the study showed that the individuals with O+ blood type were the largest group of convalescent plasma donors (Figure 3) in the southern zone of Odisha, India. 

**Figure 4:**
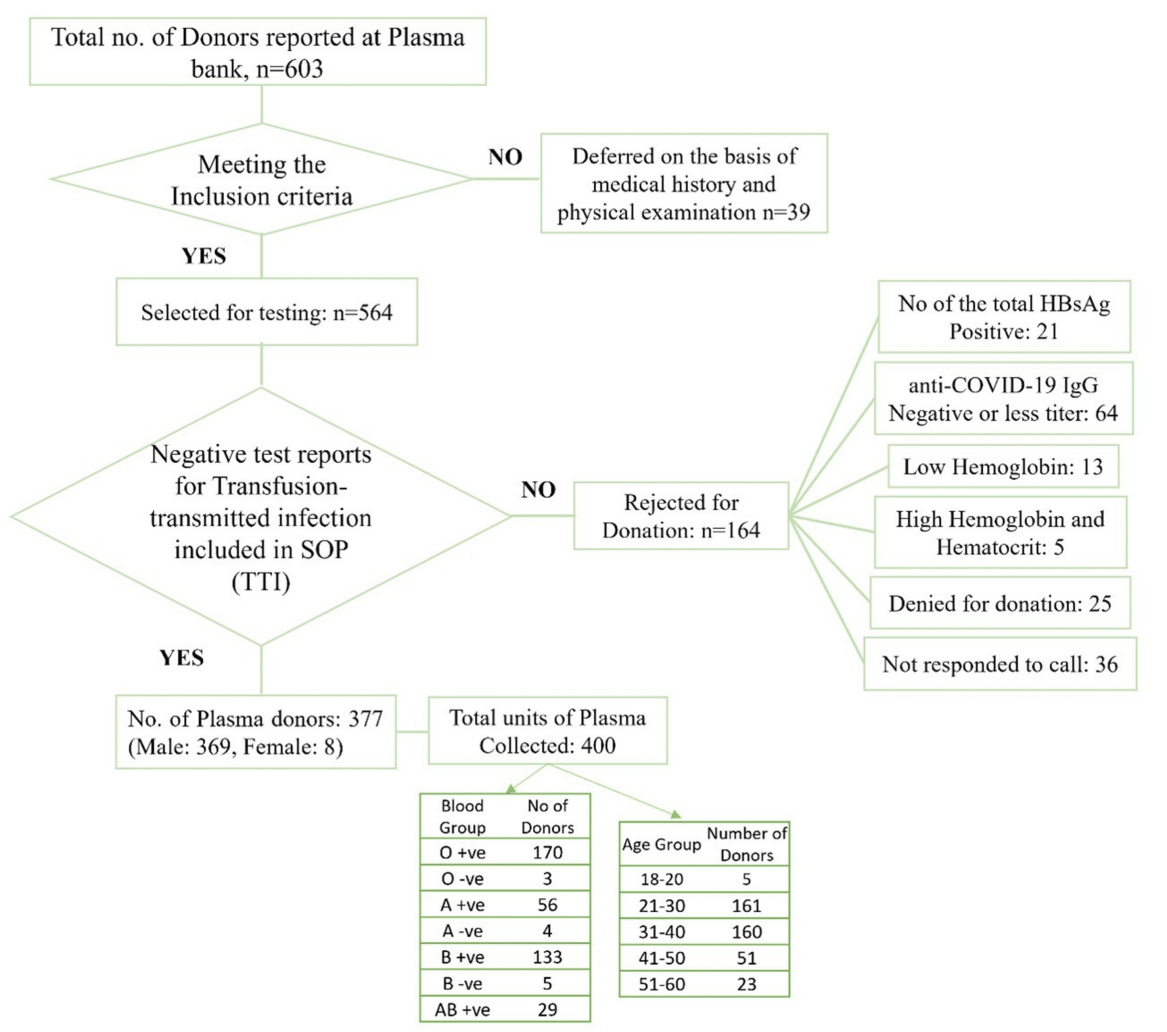
Schematic flow chart represents summary of donors based on inclusion criteria SOP - standard operating procedures

Following this, there are donors from B+ve, A+ve, and AB+ve blood groups. There was the least number (approximately 3%) of donors reported with Rh-ve blood groups. The summary of donors is listed in Figure 4.

The primary objective of this study was to manage and promote repetitive convalescent plasma donation by single donors to meet the crisis of Convalescent Plasma during the global emergency. The repetitive convalescent plasma donation was collected from 14 donors with regular examination of anti-COVID IgG antibody titer after two consecutive donations within a four-week interval (Table 2). The management of repeat convalescent plasma donation resulted in 37 repeat donations from 14 male donors. A single donor contributed to four repeat convalescent plasma donations. Following this, two male donors had donated convalescent plasma five times. We also measured the level of anti-COVID IgG antibody titer of every repeat convalescent plasma donor before their repeat donation, if donated after four weeks. We reported the repeat donation from ten donors who contributed twice after screening for the first antibody titer. The antibody titer had decreased by 8-53% during the second screening in those who donated twice after the first screening. Similarly, the antibody titer was further decreased by 90% during the third screening for the two donors who donated the convalescent plasma five times within a period of five months (Table 2).

**Table 2:** Detailed list of repeat convalescent plasma donors (mild to moderate) with their respective anti-COVID IgG antibody titer values

Sl. No	Gender	Age	1st titer	No. of repeat convalescent plasma donation after 1st Titer	2nd Titer	% Decrease in antibody titer between first and second screening	No. of repeat convalescent plasma donation after second titer	Third titer	% Decrease in antibody titer between first and third screening	No. of repeat convalescent plasma donation after third titer	Total no of repeat convalescent plasma donations
1	Male	31	9.4	2	6.91	26.49	2	4.43	89.36	1	5
2	Male	34	7.78	2	5.58	28.28	2	4.83	87.15	1	5
3	Male	37	6.53	2	3.49	46.55	2	Did not come for donation	Did not come for donation	Did not come for donation	4
4	Male	32	7.04	2	5.69	19.18	1				3
5	Male	35	7.56	2	6.42	15.08	0				2
6	Male	38	4.8	2	3.82	20.42	0				2
7	Male	33	3.04	2	2.37	22.04	0				2
8	Male	31	5.08	2	4.31	15.16	0				2
9	Male	33	4.57	2	4.19	8.32	0				2
10	Male	34	4.48	2	3.88	13.39	0				2
11	Male	30	2.61	2	1.97	24.52	0				2
12	Male	37	8.52	2	6.15	27.82	0				2
13	Male	35	3.66	2	2.5	31.69	0				2
14	Male	37	2.97	2	1.39	53.20	0				2

**Figure 5:**
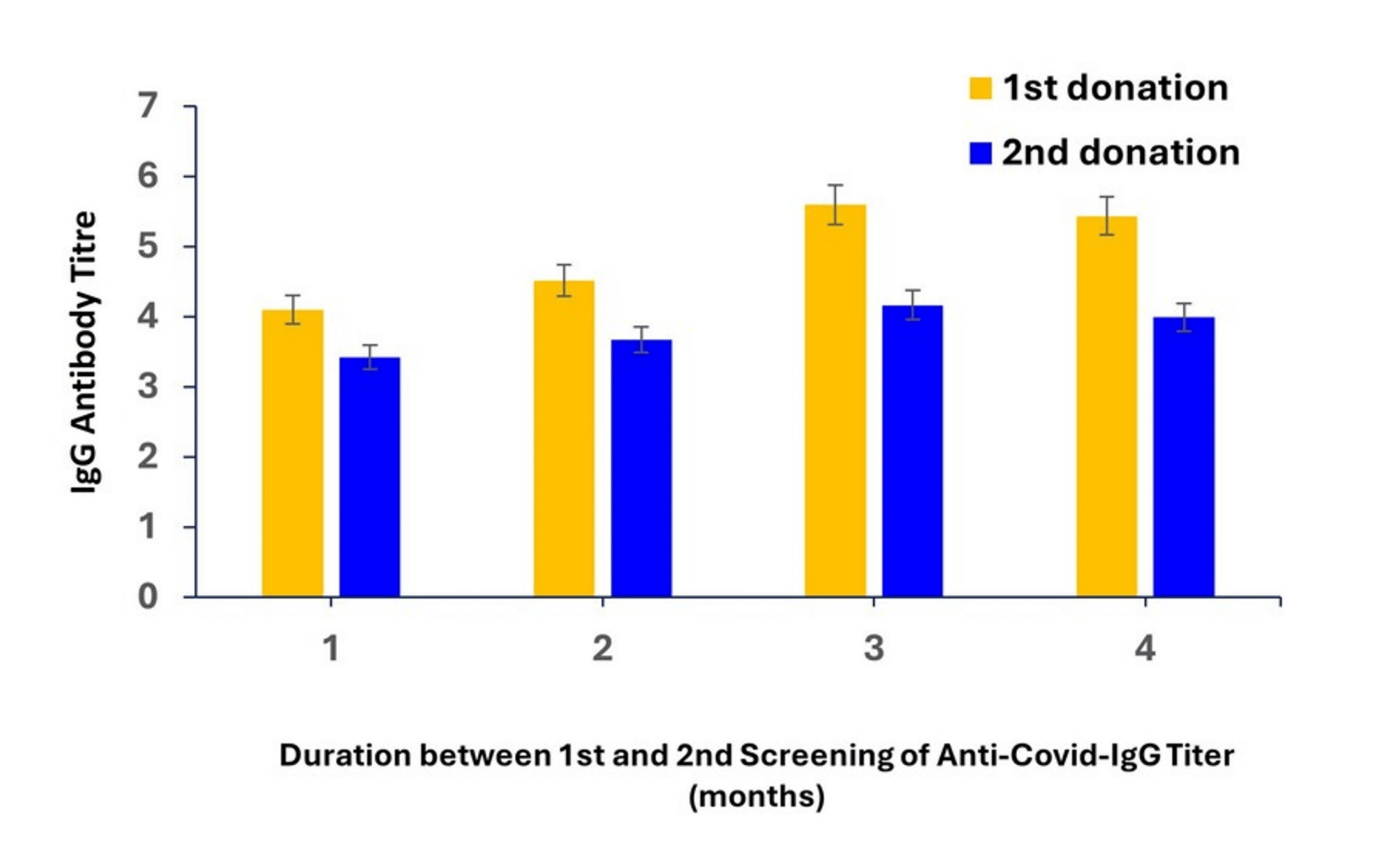
The increasing time interval between the two screening phases leads to a lowering of the antibody titer of the corresponding donors

The IgG antibody level was measured in COVID-19 patients after their complete recovery. The first screening of the anti-COVID IgG Antibody level in the recovered patients was performed after 28 days of recovery. Successful plasmapheresis donors will be requested to repeat the donation at least two weeks after the first plasma donation. The data shown in Figure 5 indicates the decreasing level of antibody titer in the two screening intervals.

This study also evaluated that the decrease in antibody levels can be directly correlated with the time interval of screening. As per the records we found, the maximum decline in IgG level (55%) was between the first and second screening among the patients who donated their plasma at an interval of 159 days (nearly five months). However, the percentage decrease in antibody level was the lowest, i.e., 17% between the two screening intervals of 30 days (one month). The significant outcome of this study reflected that the Antibody titer value gradually decreased significantly (p<0.05) from the first to the second screening with a varying time interval from one to five months.

Subsequently, the convalescent plasma units were supplied to three separate COVID-19 hospitals in Odisha. Treatment typically began with a single high-titer plasma unit, administered at a dose between 4 and 13 ml/kg (approximately 200 ml). To address demand and accommodate up to 800 recipients, each donated plasma unit was portioned into smaller 200 ml packages.

**Figure 6:**
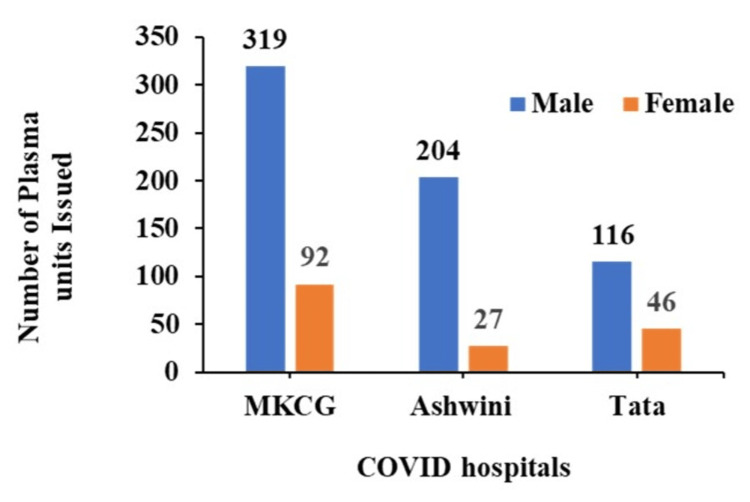
The distribution of 800 plasma units to different COVID hospitals MKCG - Maharaja Krushna Chandra Gajapati

The plasma units were issued to different COVID centers, namely MKCG dedicated COVID hospitals (MKCG-DCH) (319 Males, 92 females), Tata COVID hospital (116 Males, 46 Females), and Ashwini hospitals (204 Males, 27 Females) (Figure 6).

**Figure 7:**
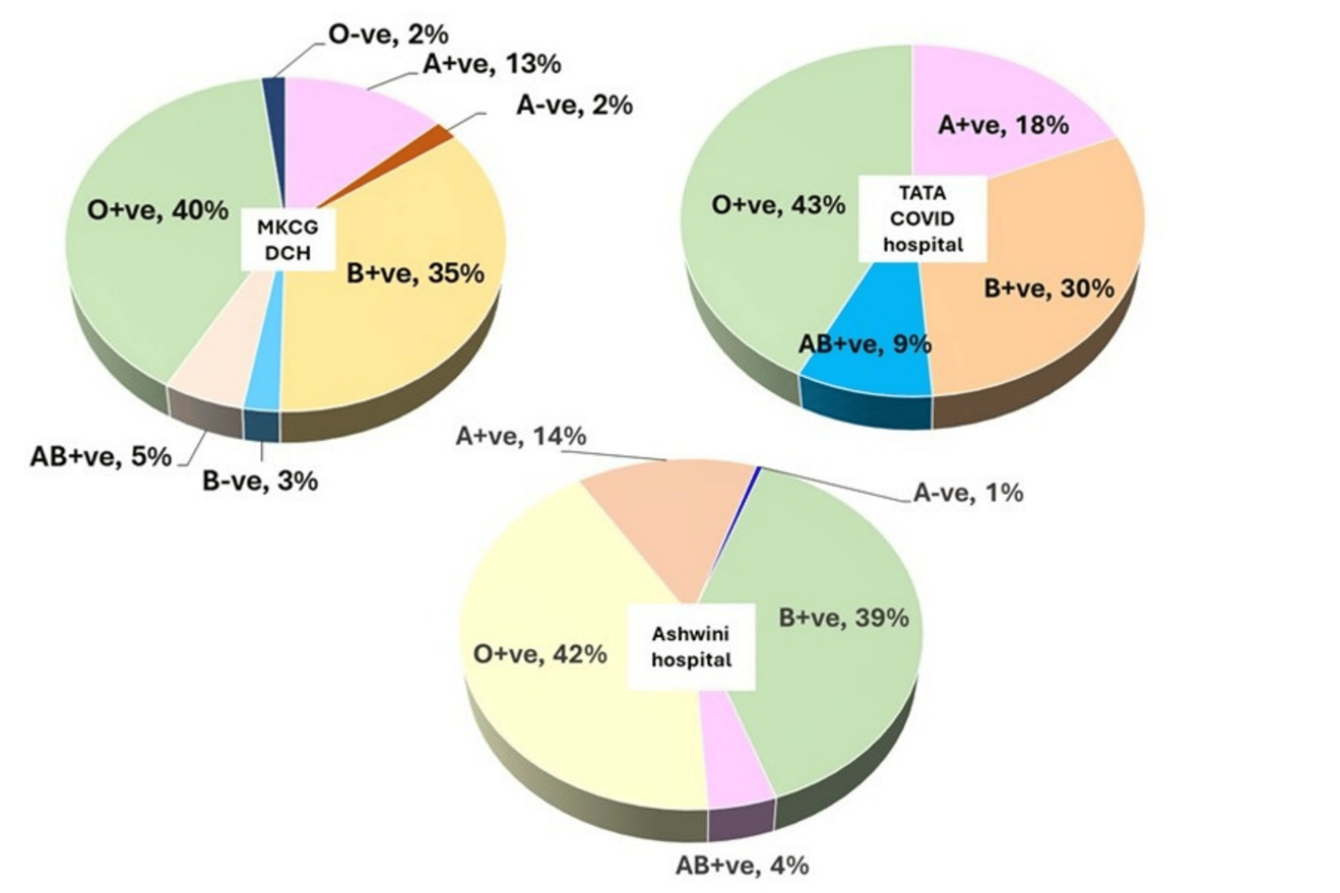
Total number of plasma units (blood group-wise) issued to different COVID hospitals MKCG DCH - Maharaja Krushna Chandra Gajapati dedicated COVID hospitals

The Convalescent plasma units were distributed to infected patients from different blood groups. The majority of the recipients at different COVID hospitals carry O+ve blood in all three COVID hospitals of southern Odisha (Figure 7), followed by B+ve and A+ve blood groups. The Rh-ve group had the fewest number of patients who had received the convalescent plasma.

**Figure 8:**
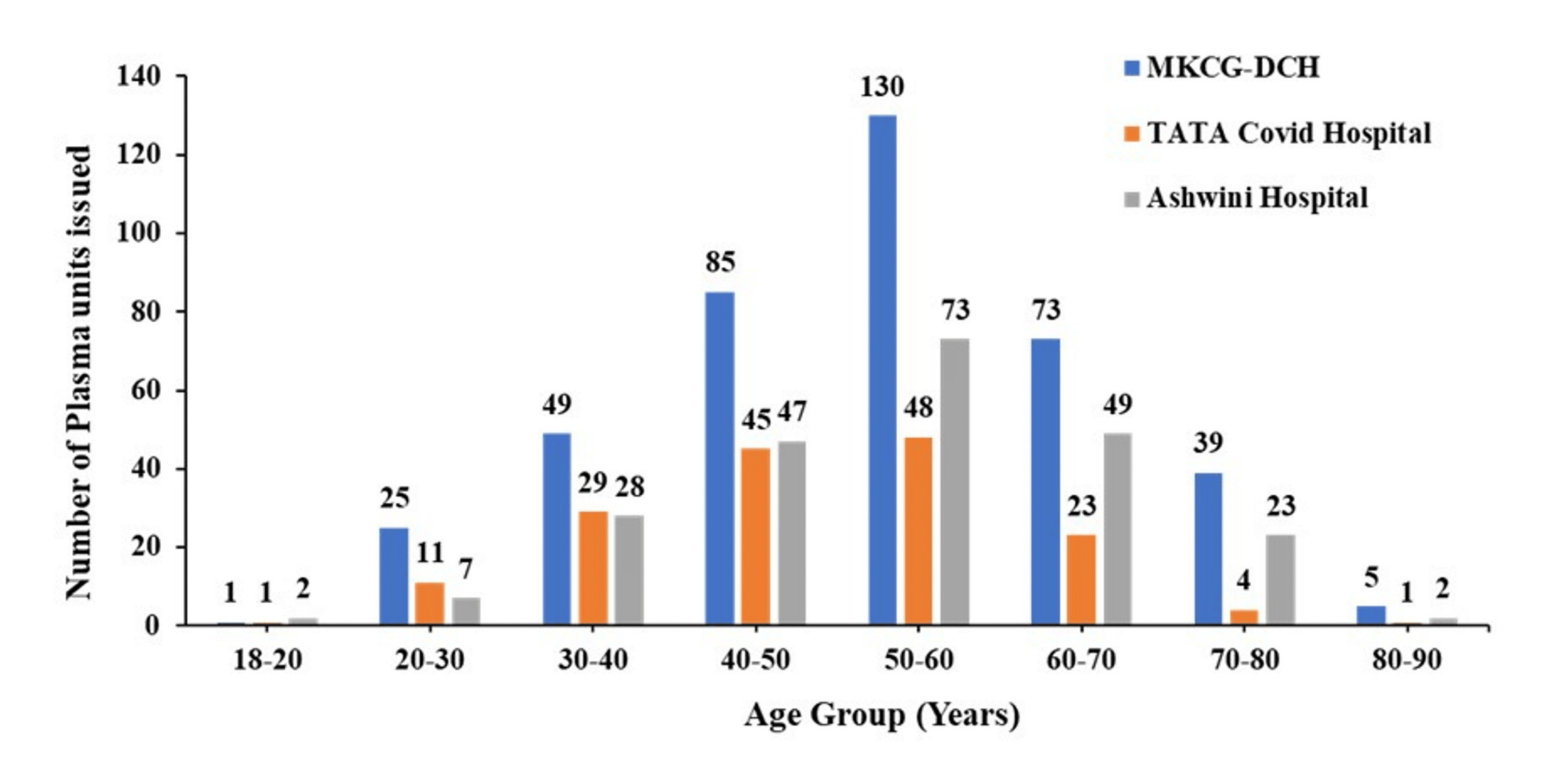
Total number of plasma units (age group-wise) supplied to different COVID hospitals MKCG DCH - Maharaja Krushna Chandra Gajapati dedicated COVID hospitals

The maximum number of plasma units is required by the age group 50-60 (30-32%), followed by 60-70 (14-21%), and 70-80 (2-10%), indicating the severity of COVID infection encountered in the patients of the same age group (Figure 8). However, this study observed that the least no. of plasma units supplied to COVID-infected patients belongs to the age groups of 18-20 and 80-90 years. This data leads to the conclusion that either patients in the younger age group (18-20 years) can recover without plasma treatment, or elderly patients (80-90 years) did not survive long enough to receive plasma therapy.

## Discussion

The COVID-19 pandemic resulted in a worldwide health emergency, marked by a scarcity of effective treatments and the lack of established prophylactic measures for those exposed to SARS-CoV-2. The potential therapeutic benefit of CP is thought to be primarily dependent on the ability of antibodies present in the plasma to neutralize SARS-CoV-2 and block infection, although other mechanisms of therapeutic benefit are possible [13]. Convalescent plasma was fast to implement, potentially provided benefits, and had a good safety profile. Previous studies also reported that despite variability in donor titer, a majority of convalescent plasma recipients showed a significant increase in antibody levels post‐transfusion during the treatment [14]. To meet the crisis of convalescent plasma as an emergency option to combat COVID-19, voluntary donor management was a major challenge. A team of doctors and health workers was engaged in patient counseling, convincing them of the importance of convalescent plasma during that crucial period. Over repetitive phone calls and requests, it was possible to motivate 603 donors to report to the Plasma Bank of Southern Odisha at MKCG, Ganjam, for voluntary donation after COVID-19 recovery.

The donors were screened for physical examination and medical history as per the SOP designed for the collection of convalescent plasma. Several donors were deferred based on the inclusion criteria, followed by their medical history. Out of them, only 564 donors were eligible for donation, waiting for their laboratory tests. Further, in the second round of screening of laboratory tests, only 377 donors were found suitable for convalescent plasma donation. The majority of the deferred category includes anti-COVID IgG-negative results during the laboratory investigations. During the COVID-19 pandemic, encouraging convalescent patients to donate plasma prior to discharge posed significant challenges. Many discharged individuals were ineligible due to exclusion criteria, and among those who qualified, only a small fraction consented to donate. A notable concern among prospective donors was the fear of potential reinfection when visiting healthcare facilities, including plasma collection centers.

During this crucial period of the pandemic, the scarcity of convalescent plasma donors was a major challenge to meet the demand. We encouraged donors to repeat donations to meet the demand at that time. There were so many limiting factors for CP donation. It was also observed that the fear factor of contracting the disease was a major barrier in encouraging COVID-19-recovered patients to donate plasma. The scarcity of convalescent plasma donation was associated with several factors; such as (i) fearful situation in revisiting the hospital setting and chances of reinfection, (ii) anxiety about becoming more susceptible to COVID-19 infection by plasma donation, (iii) a myth related to a decrease in antibody level upon plasma donation which might weaken the immune system.

This study presented the majority of male donors (n=369) during the procedure, highlighting their involvement in external or household activities. Additionally, the least contribution of female donors also emphasized the strict inclusion criteria on weight and nulliparous females. Considering the age groups, this cross-sectional study identified that most plasma donations came from respondents aged 21-30 years, particularly males with blood group O Rh D positive [15].

The primary finding of this study represents the decreasing antibody titer following repeated consecutive convalescent plasma donation. IgG was a crucial marker during the middle and late stages of infection. The efforts of the medical team in managing multiple donations from single donors in collecting repeat convalescent plasma were a major achievement of this study. The evaluation of the antibody titer for the donors with five consecutive donations represented the decreasing level of anti-COVID-IgG level up to the third test, which was within 159 days after infection. Most long-term studies have observed that specific IgG levels for SARS-CoV-1 and MERS-CoV gradually decreased over time, typically during follow-up periods of at least one year [16]. Other studies have suggested that the protective effect may only last one to two years following a coronavirus infection. Chen et al. conducted a 100-day follow-up of COVID-19 patients and observed a significant decline in IgG levels three to four months after the onset of symptoms [17]. Our findings for the repeat plasma donors aligned with their results. It showed a gradual decrease in IgG levels over time, with the most significant decline occurring within the first one to five months after discharge. This result was further supported by previous studies, which observed a decrease in IgG antibody titer up to 12 months of examination [18]. 

The distribution of convalescent plasma to different COVID hospitals indicates that the O Rh D positive group is more susceptible to infection in Odisha. The previous data in this study for repetitive convalescent plasma donation also align with these results regarding the severity of infection among O-positive blood group types. However, we did not find any donor or recipient for the AB-negative blood group at any of the designated COVID hospitals included in this study (among 800 patients). Additionally, we also found a limited number of recipients with Rh-ve blood group, suggesting their lower risk of infection during COVID-19. These results were consistent with previous findings by Kim et. al [19].

This study has a few limitations. First, it is a cross-sectional study to assess anti-SARS-CoV-2 IgG antibody levels in repetitive convalescenct plasma donors. Second, it was conducted in a small sample size as the use of convalescent plasma had suddenly dropped by the ICMR, Government of India, on 17th May 2021 due to its inefficient outcome.

## Conclusions

Convalescent plasma (CP) therapy provides immediate immunity by transferring antibodies from recovered patients. It may improve symptoms, reduce oxygen requirement and ventilation needs, and lower mortality if given to moderate to severe COVID-19 patients. CP is most effective within 7 days of infection at the right dose and titre. This study found that antibody levels decline over repeated donations, with the greatest drop by the second screening within four months. Donor deferrals due to eligibility criteria also limited convalescent plasma availability. These findings help guide COVID-19 prevention, treatment timing, and vaccine strategies.
